# Alveolar soft part sarcoma: progress toward improvement in survival? A population-based study

**DOI:** 10.1186/s12885-022-09968-5

**Published:** 2022-08-15

**Authors:** Tomohiro Fujiwara, Eiji Nakata, Toshiyuki Kunisada, Toshifumi Ozaki, Akira Kawai

**Affiliations:** 1 Department of Orthopaedic Surgery, Okayama University Graduate School of Medicine, Dentistry, and Pharmaceutical Sciences, 2-5-1 Shikata-cho, Kita-ku, Okayama, Japan; 2 Department of Musculoskeletal Oncology, National Cancer Center Hospital, 5-1-1 Tsukiji, Chuo-ku, Tokyo, Japan

**Keywords:** Alveolar soft part sarcoma, Survival, Surgery, Chemotherapy, Pazopanib

## Abstract

**Background:**

Alveolar soft part sarcoma (ASPS) is a rare histological subtype of soft-tissue sarcoma, which remains refractory to conventional cytotoxic chemotherapy. We aimed to characterize ASPS and investigate whether the oncological outcome has improved over the past decade.

**Methods:**

One hundred and twenty patients with newly diagnosed ASPS from 2006 to 2017, identified from the Bone and Soft-Tissue Tumor Registry in Japan, were analyzed retrospectively.

**Results:**

The study cohort comprised 34 (28%) patients with localized ASPS and 86 (72%) with metastatic disease at presentation. The 5-year disease-specific survival (DSS) was 68% for all patients and 86% and 62% for localized and metastatic disease, respectively (*p* = 0.019). Metastasis at presentation was the only adverse prognostic factor for DSS (hazard ratio [HR]: 7.65; *p* = 0.048). Patients who were > 25 years (80%; *p* = 0.023), had deep-seated tumors (75%; *p* = 0.002), and tumors > 5 cm (5–10 cm, 81%; > 10 cm, 81%; *p* < 0.001) were more likely to have metastases at presentation. In patients with localized ASPS, adjuvant chemotherapy or radiotherapy did not affect survival, and 13 patients (45%) developed distant metastases in the lung (*n* = 12, 92%) and brain (*n* = 2, 15%). In patients with metastatic ASPS (lung, *n* = 85 [99%]; bone, *n* = 12 [14%]; and brain *n* = 9 [11%]), surgery for the primary or metastatic site did not affect survival. Prolonged survival was seen in patients who received pazopanib treatment (*p* = 0.045), but not in those who received doxorubicin-based cytotoxic chemotherapy. Overall, improved DSS for metastatic ASPS has been observed since 2012 (5-year DSS, from 58 to 65%) when pazopanib was approved for advanced diseases, although without a statistically significant difference (*p* = 0.117).

**Conclusion:**

The national study confirmed a unique feature of ASPS with frequent metastasis to the lung and brain but an indolent clinical course. An overall trend toward prolonged survival after the introduction of targeted therapy encourages continuous efforts to develop novel therapeutic options for this therapeutically resistant soft-tissue sarcoma.

**Supplementary Information:**

The online version contains supplementary material available at 10.1186/s12885-022-09968-5.

## Introduction

Alveolar soft part sarcoma (ASPS), first described by Christopherson et al. in 1952 [1], is a rare histological subtype of sarcoma, accounting for approximately 0.5–1% of all soft-tissue sarcomas [2, 3]. ASPS primarily affects younger patients, with a peak incidence age of 15–35 years [2, 3], and female predominance is well documented [1, 4–6]. Indeed, Surveillance, Epidemiology, and End Results Program data analysis revealed that 72% of patients were aged < 30 years, and 58% were females [3]. ASPS commonly originates from deep soft tissues of the extremities [1, 7–9], predominantly the lower extremities, followed by the trunk, but may also arise from the head and neck, internal organs, tongue, and bone [5, 7, 10–15]. Molecular studies have identified a specific translocation, der (17)t(X;17)(p11.2;q25), which results in *ASPSCR1-TFE3* gene fusion [6]. Clinically, ASPS presents as a slow-growing, painless mass with high vascularity [6], carries a high rate of early distant metastasis [16, 17], and is characterized by resistance to conventional cytotoxic chemotherapy [18].

Although ASPS is refractory to conventional cytotoxic chemotherapy, this tumor is a targetable sarcoma [19]. The *ASPSCR1-TFE3* fusion gene leads to aberrant transcription of hypoxia-inducible factor 1α (HIF-1α), which upregulates proangiogenic factors, including vascular endothelial growth factor (VEGF) and hepatocyte growth factor receptor (MET/HGFR), and induces immunosuppression in the tumor microenvironment [20, 21]. These molecular features have encouraged the exploration of targeted therapy, such as antiangiogenic drugs and immune-stimulating therapy [19]. Anti-VEGF receptor tyrosine-kinase inhibitors, such as pazopanib, regorafenib, axitinib, and cediranib have shown modest antitumor activity [19, 22–24], among which pazopanib has been approved for the second-line or later treatment of patients with advanced soft-tissue sarcoma in Japan since 2012 [25]. The immune checkpoint inhibitors against PD-1, PD-L1, and CTLA-4, have also shown modest activity in several clinical trials for soft-tissue sarcomas [26, 27]. In a phase II axitinib (anti-VEGF receptor tyrosine-kinase inhibitor) plus pembrolizumab (anti-PD-1 inhibitor) trial, a clinical benefit was observed in 73% of patients with ASPS. Of 11 evaluable patients with ASPS, 6 (55%) achieved a partial response and 2 (18%) achieved stable disease [28]. Clinical trials of the anti-PD-1 inhibitor nivolumab (NCT03277924) and anti-PD-L1 inhibitor atezolizumab (NCT03277924) are currently under investigation for advanced ASPS. However, the overall survival outcome in patients with ASPS and the nationwide impact of the introduction of these novel therapies remain unknown.

Therefore, the purpose of this study was to characterize ASPS using the Bone and Soft-Tissue Tumor Registry (BSTTR) Database in Japan and investigate whether the oncological outcomes have improved since the approval of the targeted drugs. To clarify the effect of evolution in the treatment modality, we conducted our analyses using two cohorts comprising patients with localized and metastatic ASPS.

## Patients and methods

### Data source

The primary data source for this study was the BSTTR Database in Japan. This database is a nationwide organ-specific cancer registry for bone and soft-tissue tumors, which was headquartered in the National Cancer Center Hospital and funded by the Japanese Orthopaedic Association (JOA). Data were collected from 89 JOA-certified hospitals, in which the registration of data is mandatory, and other hospitals, in which the participation of data registration is voluntary. The data are updated annually. This study was approved by the Institutional Review Board of the JOA.

### Study population

Patients with a diagnosis of ASPS were searched in the registry from 2006 to 2017. A total of 181 patients were identified in the database. The inclusion criteria were patients who were newly diagnosed with pathological confirmation. Thus, we excluded 45 patients who were registered after previous treatment elsewhere and 1 patient who was not histologically diagnosed. Fifteen patients without any data required for analyses were also excluded.

### Outcomes and covariates

The primary outcome of the study was disease-specific survival (DSS). The following details were extracted from the database: basic demographics (age, sex, status at the first visit [newly diagnosed or referral after initial treatment elsewhere], and date of referral); tumor-related information (date of diagnosis, method of diagnosis [pathologically or clinically diagnosed], tumor grade, tumor site, tumor depth, metastasis at the time of diagnosis, and site of metastasis); treatment-related information (surgery for primary site, surgery for metastatic site, use of systemic therapy and/or radiotherapy, and regimen of systemic therapy); and information regarding the outcome at the last follow-up, including oncological outcome. Patients were restaged according to the American Joint Committee on Cancer (AJCC) TNM staging system, eighth edition [29]. The surgical margin was registered according to the system by Enneking et al. [30] as radical, wide, marginal, or intralesional margins.

### Statistical analysis

The Kaplan–Meier method was used to estimate the DSS and metastasis-free survival (MFS), and the differences were calculated using the log-rank test. DSS was defined as the period between the date of diagnosis and tumor-related death. Patients who died of other causes were considered as censored at the time of death. MFS was defined as the period between the date of diagnosis and the date when the distant metastasis was found. Correlations between clinicopathological variables and localized/metastatic disease were compared using the chi-square test or Fisher’s exact test. The threshold for statistical significance was *p* < 0.05. All analyses were conducted using SPSS version 23 (SPSS, Inc., Chicago, IL).

## Results

### Clinical characteristics and survival outcomes for all patients

The study cohort comprised 120 patients with newly diagnosed ASPS. The demographic and tumor characteristics are summarized in **Table ****1**. The median age of the patients was 27 years (interquartile range [IQR], 21–34 years). A female predominance was observed in this cohort; females were near twice as many (*n* = 78; 65%) as males (*n* = 42; 35%). The most frequent site of involvement was the lower extremity (*n* = 74; 62%), followed by the trunk (*n* = 34; 28%), upper extremity (*n* = 11; 9%), and head and neck (*n* = 1; 1%). The median tumor size was 7.0 cm (IQR, 5.4–10.0 cm), and most tumors were located deep to the fascia (*n* = 113; 94%). Clinically, 34 (28%) patients presented with localized disease and 86 (72%) with metastatic disease. According to the AJCC staging, 16 (13%) presented with stage II disease, 13 (11%) with stage IIIA, 5 (4%) with stage IIIB, and 86 (72%) with stage IV. The mean follow-up period was 31.5 months (range, 1–128 months).

**Table 1:** Clinical characteristics and univariate analysis of predictors for DSS

Variable	N	%	5-year DSS	*p* value
Total	120	100	68%	–
Age (median: 27 years)				0.630
≤ 25 years	50	42%	73%	
> 25 years	70	58%	65%	
Sex				0.429
Male	42	35%	62%	
Female	78	65%	72%	
Tumor site				0.517
Lower extremity	74	62%	72%	
Upper extremity	11	9%	80%	
Trunk	34	28%	63%	
Head and neck	1	1%	0%	
Tumor depth				0.922
Superficial	7	6%	83%	
Deep	113	94%	68%	
Tumor size (median: 7.0 cm)				0.200
≤ 5 cm	27	23%	94%	
> 5 cm, ≤ 10 cm	67	56%	63%	
> 10 cm	26	22%	65%	
Stage (AJCC 8th)				0.061
II	16	13%	100%	
IIIA	13	11%	100%	
IIIB	5	4%	0%	
IV	86	72%	62%	
Metastasis at diagnosis				0.019
Yes	86	72%	62%	
No	34	28%	86%	
Surgery				0.252
Yes	86	72%	74%	
No	34	28%	52%	
Radiotherapy				0.147
Yes	16	13%	63%	
No	104	87%	69%	
Systemic therapy				0.241
Yes	55	46%	61%	
No	65	54%	77%	

**Fig. 1:**
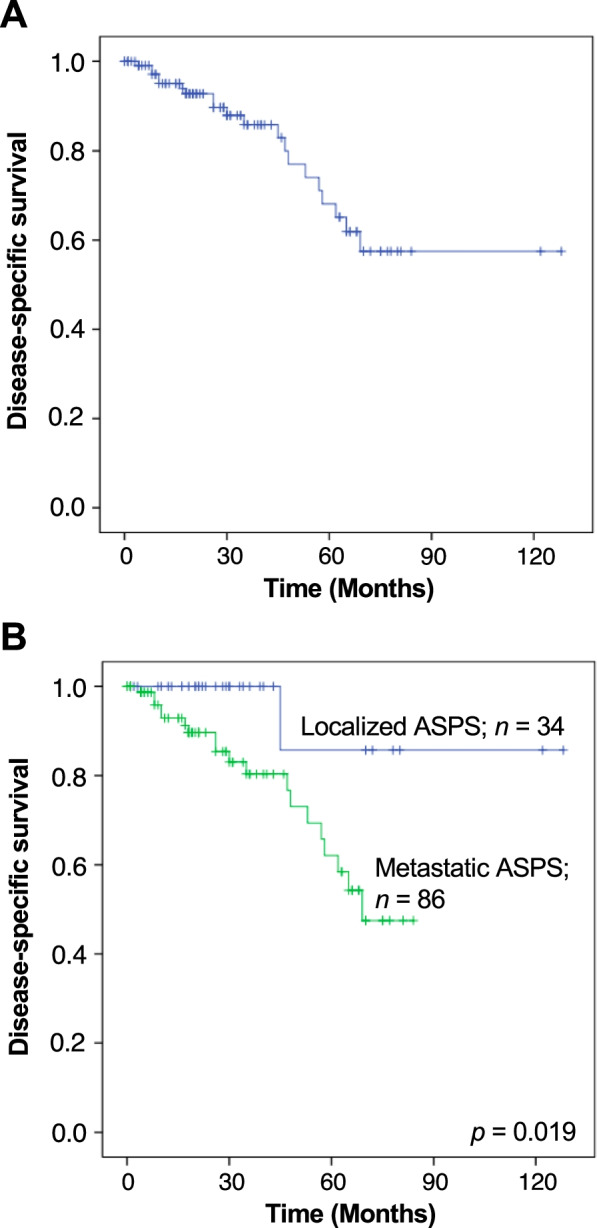
Kaplan–Meier curves showing the disease-specific survival for all patients studied (**A**) and localized versus metastatic disease at the time of diagnosis (**B**)

Overall, the 3- and 5-year estimated DSSs were 86% and 68%, respectively (**Fig. ****1****A**). The univariable analysis revealed that the presence of metastasis at the time of diagnosis was the only factor significantly associated with worse DSS (*p* = 0.019; Table 1) (present: HR, 7.65; 95% confidence interval [CI], 1.02–57.28 versus absent: HR, 1; *p* = 0.048). The clinical characteristics according to the presence of distant metastasis at diagnosis are shown in Table 2. Patients who were older than 25 years (80%; *p* = 0.023), had deep-seated tumors (75%; *p* = 0.002), and had tumors > 5 cm (5–10 cm, 81%; > 10 cm, 81%; *p* < 0.001) were more likely to have metastatic disease at the time of diagnosis (Table 2). The treatment patterns and survival outcomes of patients with localized versus metastatic disease were analyzed separately.

**Table 2:** Clinical characteristics of patients with localized versus metastatic ASPS

Variable	Localized ASPS (*n* = 34)		Metastatic ASPS (*n* = 86)		*p* value
	N	%	N	%	
Age					0.023
≤ 25 years	20	40%	30	60%	
> 25 years	14	20%	56	80%	
Sex					0.289
Male	9	21%	33	79%	
Female	25	32%	53	68%	
Tumor site					0.520
Lower extremity	19	26%	55	74%	
Upper extremity	5	46%	6	55%	
Trunk	10	29%	24	71%	
Head and neck	0	0%	1	100%	
Tumor depth					0.002
Superficial	6	86%	1	14%	
Deep	28	25%	85	75%	
Tumor size					< 0.001
≤ 5 cm	16	59%	11	41%	
> 5 cm, ≤ 10 cm	13	19%	54	81%	
> 10 cm	5	19%	21	81%	

### Treatments and survival outcomes in patients with localized ASPS

Thirty-four patients presented with localized ASPS at the time of diagnosis. Most patients (*n* = 29; 85%) underwent surgical excision. Patients without surgical treatment (*n* = 5) were excluded from further analyses (Table 3). The treatment approaches used for these patients primarily comprised local therapy alone (*n* = 27; 93%). Twenty-five patients received surgery alone, and two patients underwent surgical excision plus adjuvant radiotherapy. Two patients received neoadjuvant/adjuvant chemotherapy: one received neoadjuvant chemotherapy followed by surgery plus adjuvant radiotherapy and one underwent surgery followed by adjuvant chemotherapy. The surgical margins achieved were wide in 26 (90%), marginal in 3 (7%), and other (radical) in 1 (3%). No local recurrence was recorded during the study period.

**Table 3:** Univariable analysis of predictors for DSS and MFS in patients with localized ASPS

Variable	N	%	5-year DSS	*p* value	5-year MFS	*p* value
Age (median: years)				0.527		0.947
≤ 25 years	16	55%	100%		61%	
> 25 years	13	45%	80%		0%	
Sex				0.114		0.240
Male	7	24%	50%		29%	
Female	22	76%	100%		21%	
Tumor site				NA		0.567
Lower extremity	18	62%	83%		25%	
Upper extremity	4	14%	NA		50%	
Trunk	7	24%	100%		0%	
Tumor depth				0.683		0.630
Superficial	5	17%	100%		0%	
Deep	24	83%	83%		25%	
Tumor size				0.527		0.391
≤ 5 cm	13	45%	100%		76%	
> 5 cm	16	55%	56%		22%	
Type of surgery				0.076		0.082
Limb-salvage	27	93%	100%		21%	
Amputation	1	3%	0%		0%	
Unknown	1	3%	NA		0%	
Resection margin				NA		0.439
Intralesional	0	–	–		–	
Marginal	3	7%	NA		NA	
Wide	26	90%	100%		19%	
Other (radical)	1	3%	0%		0%	
RT				NA		0.689
No	27	93%	86%		19%	
Adjuvant^a^	2	7%	NA		NA	
Chemotherapy				0.683		0.014
No	27	93%	83%		20%	
Neoadjuvant/adjuvant^b^	2	7%	100%		0%	

^a^Adjuvant use, *n* = 2

^b^Neoadjuvant use, *n* = 1; adjuvant use, *n* = 3; *NA* Not available

The 3- and 5-year estimated DSSs were 100% and 86%, respectively (Fig. 1B). None of the analyzed variables were significantly associated with DSS. The surgical margin, the administration of neoadjuvant/adjuvant chemotherapy, and the use of radiotherapy did not affect the DSS (Table 3).

**Fig. 2:**
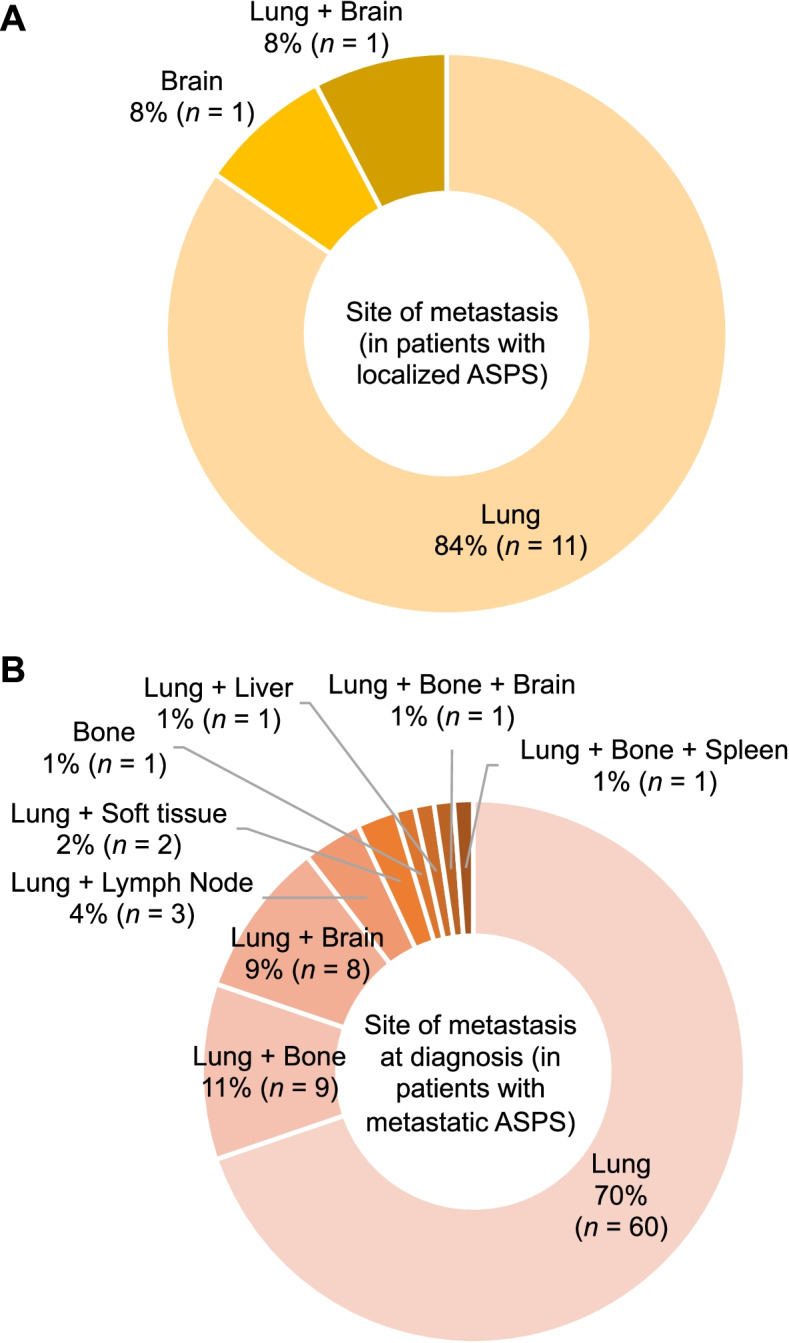
Sites of metastasis developed in patients with localized (**A**) and metastatic ASPS (**B**)

During the study period, 13 patients (45%) developed distant metastases. The sites of metastasis were the lung in 11 patients, lung + brain in 1, and brain in 1 (Fig. 2A); lung and brain metastases developed in 35% and 6% of patients with localized ASPS, respectively. The 3- and 5-year estimated MFSs were 55% and 18%, respectively. The use of chemotherapy was negatively associated with MFS (*p* = 0.014; Table 3). Neither the surgical margin nor the use of adjuvant radiotherapy affected the MFS.

### Treatments and survival outcomes in patients with metastatic ASPS

Eighty-six patients presented with metastatic ASPS at the time of diagnosis. The sites of metastasis at diagnosis were lung in 60 patients, lung + bone in 9, lung + brain in 8, lung + lymph node in 3, lung + soft-tissue in 2, bone in 1, lung + liver in 1, lung + bone + brain in 1, and lung + bone + spleen in 1 (Fig. 2B): lung metastases were observed in 85 patients (99%), bone metastases in 12 (14%), and brain metastases in 9 (11%).

Local treatment of the primary site of the tumor was performed in 59 of 86 patients with metastatic ASPS (69%): surgery alone in 55 patients (64%), surgery + radiotherapy in 2 (2%), and radiotherapy alone in 2 (2%). The surgical margins achieved in patients who underwent surgical excision were wide in 51 patients (90%), marginal in 4 (7%), intralesional in 1 (2%), and unavailable in 1 (2%). Surgery for metastasis was performed in 11 patients (13%), radiotherapy was administered palliatively in 9 (11%), and systemic treatment was performed in 48 (56%): conventional cytotoxic chemotherapy in 14 (16%), targeted therapy in 19 (22%), conventional cytotoxic chemotherapy + targeted therapy in 11 (13%), and unknown regimen in 4 (5%). Regimens of the systemic treatment are summarized in Supplementary Table 1. The doxorubicin (DOX)-based cytotoxic chemotherapy regimens were administered in 23 of 25 patients who received conventional cytotoxic chemotherapy (92%). Pazopanib was administered in 27 of 30 patients (90%) who received targeted therapy. The proportion of patients who underwent systemic treatments for metastatic ASPS has significantly increased since 2012 (49% versus 62%; *p* = 0.002), when the use of pazopanib was approved by the government, and, accordingly, conventional cytotoxic chemotherapy was performed less frequently (41% versus 19%; *p* = 0.002).

**Fig. 3:**
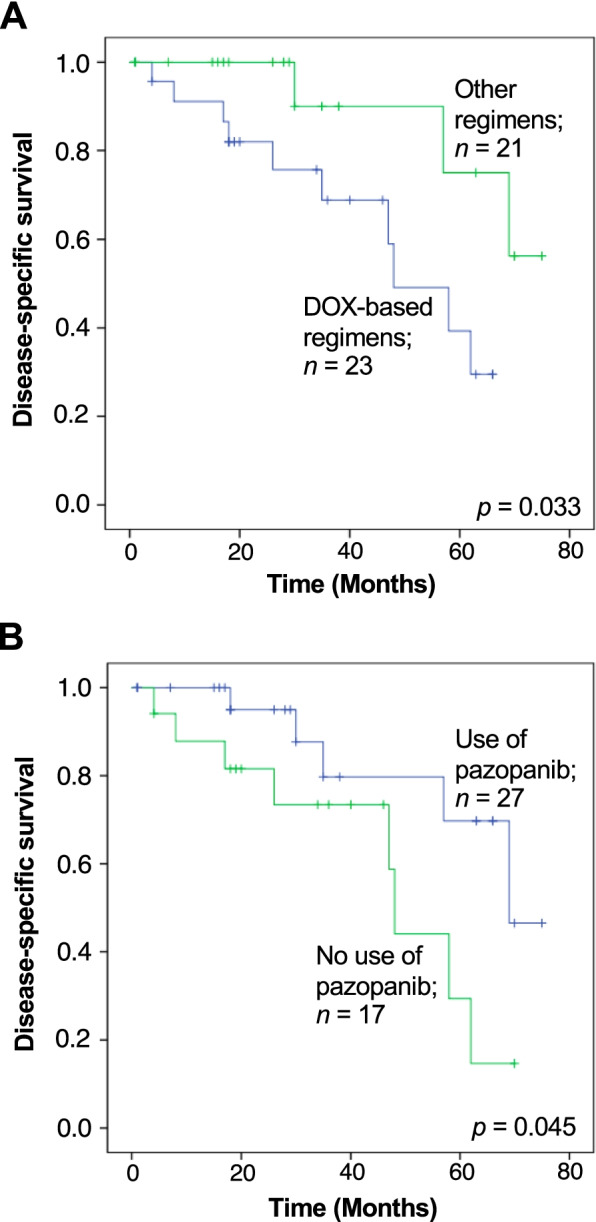
Kaplan–Meier curves showing the disease-specific survival in patients who received systemic treatments for metastatic ASPS, stratified by the use of doxorubicin (DOX) (**A**) and pazopanib (**B**)

**Fig. 4:**
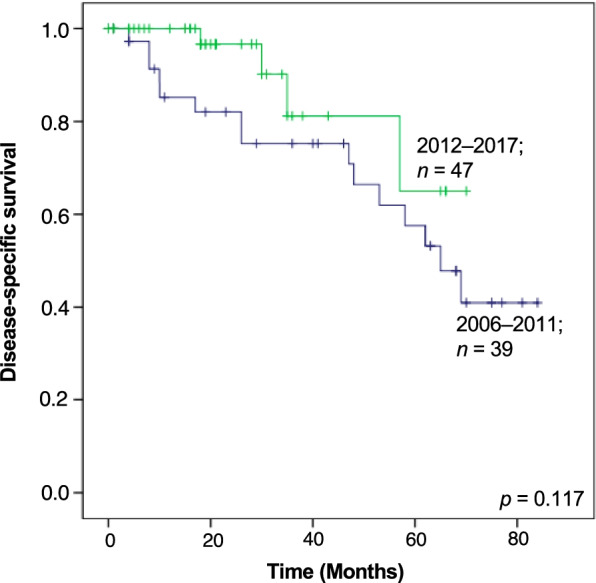
Kaplan–Meier curves showing the disease-specific survival in patients with metastatic ASPS, stratified by the era of treatments; 2006–2011 versus 2012–2017

The 3- and 5-year estimated DSSs were 80% and 62%, respectively (Fig. 1B). The median survival period in patients with metastatic ASPS was 69 months. The univariable analysis revealed that the tumor depth at the primary site was associated with survival outcome; deep-seated ASPS was significantly associated with worse DSS (5-year DSS, 63%; *p* = 0.006). Surgical resection of the primary (*p* = 0.559) or metastatic site (*p* = 0.143), receipt of radiotherapy (*p* = 0.614), and administration of systemic therapy (*p* = 0.470) did not affect the DSS (Table 4). Among the 44 patients with available data on the type of systemic drug, we observed no significant difference in DSS according to the type of therapeutic drug. The 5-year DSS was 34%, 66%, and 55% in patients who received cytotoxic chemotherapy, targeted therapy, and cytotoxic chemotherapy + targeted therapy, respectively (*p* = 0.535). In terms of the therapeutic regimen, patients who received DOX-based cytotoxic chemotherapy regimens had significantly inferior DSS; the 5-year DSSs were 39% and 75% in patients with and without DOX-based cytotoxic chemotherapy regimens, respectively (*p* = 0.033; Fig. 3A). Of note, patients who did not receive DOX-based cytotoxic chemotherapy regimens were mostly treated with pazopanib (*n* = 20/21; 95%): monotherapy in 17 (81%) and combined therapy with other drugs in 3 (14%). Prolonged survival was seen in patients who received pazopanib treatment; the 5-year DSSs were 70% and 29% with and without pazopanib, respectively (*p* = 0.045; Fig. 3B). Overall, the median survival period in patients with pazopanib treatment was 70 months, whereas patients who received DOX-based cytotoxic chemotherapy had a median survival period of 48 months (Figs. 3A and 3B). In a comparison before and after the approval of pazopanib, we observed a trend toward superior DSS in patients who had a diagnosis and/or treatment for metastatic ASPS after 2012 (5-year DSS, 65%) compared to those before 2012 (5-year DSS, 58%; Fig. 4), although this did not reach statistical significance (*p* = 0.117).

**Table 4:** Univariable analysis of predictors for DSS in patients with metastatic ASPS

Variable	N	%	5-year DSS	*p* value
Age (median: years)				0.911
≤ 25 years	30	35%	64%	
> 25 years	56	65%	60%	
Sex				0.997
Male	33	38%	63%	
Female	53	62%	61%	
Tumor site				0.542
Lower extremity	55	64%	68%	
Upper extremity	6	7%	75%	
Trunk	24	28%	55%	
Head and neck	1	1%	0%	
Tumor depth				0.006
Superficial	1	1%	0%	
Deep	85	99%	63%	
Tumor size				0.719
≤ 5 cm	11	13%	83%	
> 5 cm, ≤ 10 cm	54	63%	54%	
> 10 cm	21	24%	76%	
Resection of the primary site				0.559
No	29	34%	51%	
Yes	57	66%	68%	
Resection of the metastatic site				0.143
No	75	87%	62%	
Yes	11	13%	67%	
RT				0.614
No	73	85%	61%	
For primary lesion	4	5%	38%	
For metastases	9	11%	73%	
Systemic treatment				0.470
No	38	44%	73%	
Conventional cytotoxic chemotherapy	14	16%	34%	
Targeted therapy	19	22%	66%	
Conventional cytotoxic chemotherapy + targeted therapy	11	13%	55%	
Unknown	4	5%	NA	
Regimen (information available; *n* = 44)				
DOX-based regimen				0.033
Yes	23	27%	39%	
No	21	24%	75%	
Use of pazopanib				0.045
Yes	27	31%	70%	
No	17	20%	29%	

*Abbreviation*: *RT* Radiotherapy, *DOX* Doxorubicin, *NA* Not available

## Discussion

Although ASPS has greater metastatic potential than other soft-tissue sarcomas, the natural history of the tumor appears to be indolent [18]. Overall, the 5-year DSSs for localized and metastatic ASPS in this study were 86% and 62%, respectively, which are comparable to the previous studies (Table 5). For localized ASPS, the 5-year survival rate of approximately 60% was reported in 1989 by Lieberman et al. [4], while similar results have been observed almost 30 years later in the more recent series [3, 17, 19, 31]. In a recent study using the National Cancer Database (USA), the 5-year overall survival (OS) was 73% in 83 patients with localized disease [17]. These data urge the innovation of more effective neoadjuvant/adjuvant therapy for localized ASPS. For metastatic ASPS, Lieberman et al. reported the 5-year OS was 22% in 1989, while a similar percentage (5-year OS, 20%) was noted in 2001 by Portea et al. [2]. Recent series have described more favorable outcomes; Flores et al. reported that the 5-year OS was 61% in 38 patients with metastatic disease [31]. The improvement in survival may be attributed to the introduction of the targeted therapies, although this should be confirmed using a larger cohort of patients. Indeed, our data show a trend toward superior DSS after the approval of pazopanib for advanced soft-tissue sarcomas.

**Table 5:** Clinicopathologic studies of ASPS

Source	No. of patients (localized/metastatic)	5-year survival			Prognostic factor	Comments
		Overall	Localized	Metastatic		
Lieberman et al., 1989 [4]	91 (69/22)	57%	60%	22%	Age, metastasis	Increased rate of metastasis at presentation as the age increases
Casanova et al., 2000 [9]	19 (15/4)	80%	91%	NA	Size	Series of pediatric patients (median age, 12 years)
Portea et al., 2001 [2]	74 (22/52)	47%	88%	20%	Metastasis	Brain metastasis in 9 of 48 patients (18.8%) with metastatic disease
Ogose et al., 2003 [5]	57 (20/37)	56%	81%	46%	Size, metastasis, bone involvement	Bone involvement at the primary site in 23%
Daigeler et al., 2008 [32]	11 (11/0)	88%	88%	–	None	Brain and lung metastasis in 3 of 3 patients (100%) who developed metastases
Ogura et al., 2012 [16]	26 (10/16)	64%	100%	37%	Size, metastasis	Median survival, 90 months
Wang et al., 2016 [3]	251 (118/108)	56%	81%	41% (surgery +), 10% (surgery-)	Age, size, metastasis, trunk, no treatment, RT without surgery	Improved OS with surgery + RT (localized) Improved OS with surgery of primary site (metastatic)
Brennan et al., 2018 [33]	22 (20/2)	100%	100%	100%	NA	Series of pediatric patients (median age, 11.5 years) 5-year EFS, 94.7% (localized disease)
Flores et al., 2018 [31]	69 (31/38)	72%	87%	61%	Age, sex, metastasis	Series of pediatric patients (< 30 years)
Hagerty et al., 2020 [17]	293 (83/172)	NA	73% (surgery +)	46% (surgery +)	Size, margin, metastasis, multimodal therapy, hospital volume	Analysis from National Cancer Database in the United States
Current study, 2022	120 (34/86)	68%	86%	62%	Metastasis	Analysis from BSTTR Database in Japan

*Abbreviations*: *OS* Overall survival, *BSTTR* Bone and Soft-Tissue Tumor Registry, *NA* Not available

For localized ASPS, complete surgical resection appears as the only curative treatment. Patients who underwent neoadjuvant/adjuvant chemotherapy (*n* = 2) or radiotherapy (*n* = 2) were limited; thus, we could not determine their efficacy in the management of localized disease. The published literature describes the resistance to conventional cytotoxic chemotherapy [2, 8, 16, 18, 34, 35] with a complete or partial remission rate of < 10% [36]. Neoadjuvant/adjuvant radiotherapy has been described to be more effective for local treatment compared to surgery alone [3, 31]. Further analyses based on the larger cohort of patients are necessary to determine the effect of neoadjuvant/adjuvant therapies. Of note, favorable local controls in the current cohort could be explained by no intralesional resection of the tumor registered in the BSTTR Database.

No curative therapy has been devised for metastatic ASPS. Although the DOX remains the standard first-line therapy for soft-tissue sarcomas [37–39], the efficacy of the DOX-based chemotherapy was not proven for metastatic ASPS. Considering the refractoriness of ASPS to conventional cytotoxic chemotherapy, targeted therapy appears as an attractive alternative. In this study, pazopanib appeared to have a possible survival benefit, with a median DSS of 70 months. Recent studies have documented the antitumor activity of pazopanib in metastatic ASPS [40–44]. Oh et al. retrospectively analyzed the outcomes of pazopanib treatment in patients with advanced ASPS (*n* = 10) and reported a median overall survival of 48 months, which was favorable compared to the other histological subtypes [42]. Moreover, Jagodzińska-Mucha et al. confirmed the long-term efficacy of sunitinib, an antiangiogenic molecule, in patients with metastatic ASPS (*n* = 15), with a median overall survival of 56 months [45]. Although the effect of sunitinib could not be evaluated in this study because of the limited number of patients treated with this drug (*n* = 1), previous studies suggest these antiangiogenics could be a putative therapeutic option in the first-line treatment of metastatic ASPS [46]. Recent studies have also demonstrated a promising role of immune checkpoint inhibitors [28, 47, 48]. However, we could not evaluate the therapeutic efficacy of the immunotherapies or targeted drugs other than pazopanib because of the limited number of patients treated with these drugs. Therefore, further studies are warranted to fully evaluate the role of these targeted therapies and immunotherapies for metastatic ASPS.

ASPS is characterized by a high incidence of brain metastasis compared to other subtypes of soft-tissue sarcoma [36]. Brain metastases are mostly observed as a component of disseminated disease [2, 5]. In this study, brain metastasis was observed in 11% of patients with metastatic ASPS and occurred in 6% of patients with localized ASPS, which was comparable to the published literatures [2, 17]. These data suggest that intracranial imaging should be added to the routine imaging studies, as mentioned in the current practice guidelines [37–39]. The effect of brain metastases on the survival of patients with metastatic ASPS remains inconclusive. In our series, brain metastases occurred as a manifestation of disseminated disease in patients with metastatic ASPS, but the presence of brain metastasis did not affect the survival compared to those with metastases at the other sites. Further study based on larger series is warranted to determine the survival impact of brain metastasis. Ogura et al. reported favorable local control of brain metastases by Gamma Knife in four patients, with a median progression-free period of 12 months [16]. These patients may be included in this patient cohort, but the data regarding the treatment for brain metastases were not available in the BSTTR Database. A recent report by Malouf et al. described low efficacy of the antiangiogenic therapies for brain metastasis of ASPS [49], indicating resistance to currently available drugs. There is a need to develop agents with high central nervous system penetrance or specific multimodal therapeutic strategies for brain metastasis of ASPS.

We acknowledge several limitations in this study. First, the BSTTR Database do not include the exact doses and toxicity of systemic therapies and radiotherapy. Thus, we could not evaluate the efficacy and safety of these therapies precisely based on the dose of administrations. Second, the reliability of our study may be challenged by the limited number of patients because of the rarity of this subtype of soft-tissue sarcoma. Multivariable analyses could not be performed because of the inadequate variables that are univariably associated with survival and the limited number of patients in localized/metastatic ASPS. Third, the follow-up period was relatively short, with a mean period of 31.5 months; this appears to be common in studies using the large databases [3, 17], and longer follow-up may lead to a decrease in disease-specific death. Fourth, the information regarding the metastatic site that was resected was unavailable if the patients had multiple metastases. Fifth, the possibility of a duplicate registration was not excluded if a patient received care at more than one hospital. However, the BSTTR is designed to automatically exclude the cases if they were referred for “second opinion/only observation after treatment in the previous hospital” to avoid duplicate reporting. Finally, we acknowledge a possibility that some patients who received care at non-JOA-certified hospitals might not be registered in the database because the registry is not mandatory for these institutions. Despite these limitations, we believe that this study is valuable in that the BSTTR Database is a nationwide sarcoma-specific registry in Japan, presenting the national trend and outcomes in the era of modern multidisciplinary treatment, which is unique compared to international trends and outcomes.

In summary, ASPS is a unique subtype of soft-tissue sarcoma, with a high metastatic rate at presentation but an indolent clinical course. Brain metastases are relatively frequent, necessitating continuous evaluation with brain magnetic resonance imaging or computed tomography in addition to routine radiological screening during the follow-up. For localized ASPS, complete resection with negative margins is the only curative therapy, and survival benefit of adjuvant chemotherapy and/or radiotherapy was not proven. For advanced ASPS, use of pazopanib was associated with prolonged survival compared to the conventional cytotoxic chemotherapy. A trend toward prolonged survival after the introduction of the targeted drugs encourages continued efforts to develop novel therapeutic options.

## Supplementary Information

**Additional file 1: Supplementary Table 1.** Regimens of systemic therapy in patients with metastatic ASPS.

## Data Availability

The datasets that support the findings of this study are available on request from the Japanese Orthopedic Association committee.

## Abbreviations

ASPS: Alveolar soft part sarcoma
DSS: Disease-specific survival
HR: Hazard ratio
HIF-1α: Hypoxia-inducible factor 1α
VEGF: Vascular endothelial growth factor
MET/HGFR: Hepatocyte growth factor receptor
PD-1: Programmed death 1
PD-L1: Programmed death 1 ligand
CTLA-4: Cytotoxic T-lymphocyte-associated antigen 4
BSTTR: Bone and Soft-Tissue Tumor Registry
JOA: Japanese Orthopaedic Association
AJCC: American Joint Committee on Cancer
MFS: Metastasis-free survival
