# Supplementary material for: Alveolar soft part sarcoma: progress toward improvement in survival? A population-based study
Source: BMC Cancer. 2022 Aug 15;22:891. doi: 10.1186/s12885-022-09968-5 (PMC9377116; doi:10.1186/s12885-022-09968-5)
Supplement: Supplementary file 1 — Additional file 1: Supplementary Table 1. Regimens of systemic therapy in patients with metastatic ASPS. [file 12885_2022_9968_MOESM1_ESM.pdf]

Supplementary Table 1. Regimens of systemic therapy in patients with metastatic ASPS

Regimen	N	%
Pazopanib	17	35%
ADR + IFO + Pazopanib	5	10%
ADR + IFO	4	8%
ADR	2	4%
ADR + VCR	1	2%
ADR + CDDP	1	2%
ADR + CPM	1	2%
ADR + IFO + DTIC	1	2%
ADR + IFO + CBDCA	1	2%
ADR + CDDP + VCR	1	2%
ADR + IFO + GEM + DOC	1	2%
ADR + IFO + Elbulin	1	2%
ADR + IFO + Sunitinib	1	2%
ADR + IFO + VCR + Pazopanib	1	2%
ADR + IFO + Elbulin + Trabectedin	1	2%
ADR + Pazopanib + Trabectedin	1	2%
IFO + VP16 + CDDP	1	2%
GEM + Sunitinib + Pazopanib	1	2%
Pazopanib + Nivolumab	1	2%
Pazopanib + Elibulin	1	2%
Unknown	4	8%
